# Predictive Value of Sp1/Sp3/FLIP Signature for Prostate Cancer Recurrence

**DOI:** 10.1371/journal.pone.0044917

**Published:** 2012-09-13

**Authors:** Roble G. Bedolla, Jingjing Gong, Thomas J. Prihoda, I-Tien Yeh, Ian M. Thompson, Rita Ghosh, Addanki P. Kumar

**Affiliations:** 1 Department of Urology, The University of Texas Health Science Center, San Antonio, Texas, United States of America; 2 Department of Pathology, The University of Texas Health Science Center, San Antonio, Texas, United States of America; 3 Department of Pharmacology, The University of Texas Health Science Center, San Antonio, Texas, United States of America; 4 Cancer Therapy and Research Center, The University of Texas Health Science Center, San Antonio, Texas, United States of America; 5 South Texas Veterans Health Care System, The University of Texas Health Science Center, San Antonio, Texas, United States of America; Baylor College of Medicine, United States of America

## Abstract

Prediction of prostate cancer prognosis is challenging and predictive biomarkers of recurrence remain elusive. Although prostate specific antigen (PSA) has high sensitivity (90%) at a PSA level of 4.0 ng/mL, its low specificity leads to many false positive results and considerable overtreatment of patients and its performance at lower ranges is poor. Given the histopathological and molecular heterogeneity of prostate cancer, we propose that a panel of markers will be a better tool than a single marker. We tested a panel of markers composed of the anti-apoptotic protein FLIP and its transcriptional regulators Sp1 and Sp3 using prostate tissues from 64 patients with recurrent and non-recurrent cancer who underwent radical prostatectomy as primary treatment for prostate cancer and were followed with PSA measurements for at least 5 years. Immunohistochemical staining for Sp1, Sp3, and FLIP was performed on these tissues and scored based on the proportion and intensity of staining. The predictive value of the FLIP/Sp1/Sp3 signature for clinical outcome (recurrence vs. non-recurrence) was explored with logistic regression, and combinations of FLIP/Sp1/Sp3 and Gleason score were analyzed with a stepwise (backward and forward) logistic model. The discrimination of the markers was identified by sensitivity-specificity analysis and the diagnostic value of FLIP/Sp1/Sp3 was determined using area under the curve (AUC) for receiver operator characteristic curves. The AUCs for FLIP, Sp1, Sp3, and Gleason score for predicting PSA failure and non-failure were 0.71, 0.66, 0.68, and 0.76, respectively. However, this increased to 0.93 when combined. Thus, the “biomarker signature” of FLIP/Sp1/Sp3 combined with Gleason score predicted disease recurrence and stratified patients who are likely to benefit from more aggressive treatment.

## Introduction

Prostate cancer (PCA) is the second leading cause of cancer-related death in men and is expected to cause 28,170 deaths in the United States in 2012 [1]. PCA generally affects men over 65 years of age but remains indolent and asymptomatic in a majority of cases. The histopathological and molecular heterogeneity of the disease makes prediction of prognosis challenging. Although PSA is the most widely used serum marker for prostate cancer, it has no accepted cut-off point with high sensitivity and specificity and often leads to false positive results [2]–[4]. Furthermore, there are currently no molecular markers that can be used to reliably predict which premalignant lesions will recur or develop into invasive PCA [2]–[6]. A valid biomarker should have the following characteristics: (i) accuracy (should not falsely predict positive or negative results); (ii) selectivity (ability to diagnose the disease during disease progression); and (iii) specificity (ability to distinguish cancerous from non-cancerous phenotype). Although PSA fulfills most of these criteria and is widely used, it is limited by its low values of specificity and selectivity [2]–[6]. Because of the growing evidence for overtreatment of prostate cancer, it is important to identify and validate new prognostic markers that will predict clinically significant prostate cancer [6]–[10]. Such markers will enable the targeted treatment of patients with aggressive tumors while avoiding unnecessary treatment and its side effects in patients with indolent disease.

**Table 1:** Characteristics of the patient population.

PATIENTS	Non-Recurrent	Recurrent
n = 64	34 (53.13%)	30 (46.88%)
Low Gleason 5−7(3+4)	28 (82.35%)	6 (23.33%)
High Gleason 7(4+3)−9	7 (17.65%)	23 (76.66%)
**AGE** #	**Non-Recurrent**	**Recurrent**
Mean	64.03	63.83
Median	66	64.5
Range	52–76	51–76
**PSA**	**At Surgery**	**At Failure**
Mean	10.04	0.341
Median	8.1	0.31
Range	1.37–54.4	0.2–1

# No significant difference between groups p = 0.82.

Research over the past decade has identified a number of biomarkers that are associated with high Gleason grade disease [7]–[13]. Previous studies from our laboratory found a correlation between expression of FLICE-inhibitory protein (FLIP) and tumor grade in human prostate cancer [13]. Specifically, we found that high-grade Gleason tumors show increased FLIP staining compared with low-grade Gleason tumors (p = 0.04) [13]. In experiments to understand the role of FLIP regulation during prostate carcinogenesis, we identified transcription factors Sp1 and Sp3 as important regulators of FLIP transcriptional activity in prostate cancer cells [13]. We further demonstrated that Sp1 trans-activates the FLIP promoter while Sp3 inhibits Sp1-mediated trans-activation, thus implicating a role for these factors during prostate carcinogenesis. However, it was not known whether any of these markers could achieve the sensitivity and specificity necessary to distinguish aggressive from indolent disease. Here, we evaluated whether the “biomarker signature” of FLIP, Sp1, and Sp3 can predict the development of prostate cancer recurrence by immunohistochemical evaluation of tissue samples obtained from patients who underwent prostatectomy as primary treatment for prostate cancer and were observed for at least 5 years with PSA measurements. We show that the combination of FLIP, Sp1, Sp3, and Gleason score is an excellent predictor of biochemical recurrence. The area under the receiver operator characteristic curve for FLIP, Sp1, and Sp3 when predicting PSA failure was 0.71, 0.66, and 0.68 respectively; however, when these three markers were combined with Gleason score the AUC increased to 0.93. This level of prediction for PSA failure suggests that this biomarker panel could be an important predictor of biochemical recurrence.

**Figure 1:**
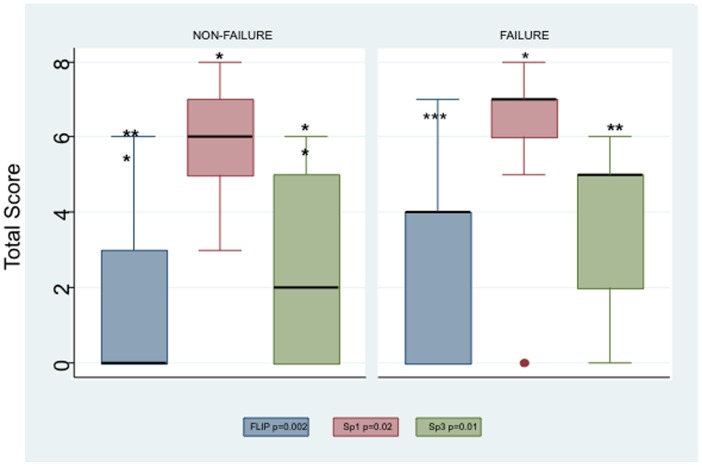
Box plots showing significant differences in mean total score for IHC of Sp1, Sp3, and FLIP between recurrent and non-recurrent cases as determined by Wilcoxon rank-sum test.

## Materials and Methods

### Patients and Tissues

We used tissues from the GU tissue repository at The University of Texas Health Science Center, San Antonio, TX for which written informed consent approval was obtained from the Institutional Review Board (Protocol number HSC 20050234H entitled Tissue bank and data base for urologic diseases) at The University of Texas Health Science Center, San Antonio, TX. These patients underwent radical prostatectomy as primary treatment for prostate cancer at University Hospital and the South Texas Veterans Health Care System, Audie Murphy Veterans Administration Hospital at San Antonio, Texas. In the current study, tissues used were from 64 unidentified patients (approved by the institutional review board of the University of Texas Health Science Center at San Antonio). Age range was from 51–76 years, median age 63 years (Table 1). Cases were classified as recurrent if PSA was detectable and increased to 0.2 ng/mL or higher, as confirmed by a second PSA test. Patients without recurrence had undetectable PSA levels or a PSA <0.2 ng/mL during at least a 60-month follow-up period after prostatectomy. Of the 64 subjects, 30 had recurrent cancer (47%) and 34 were without recurrence (53%). Gleason scores were significantly different between the two groups (p = 0.0001): 82.35% of the non-recurrent cases (PSA non-failure) had low Gleason grade [5 to 7(3+4)], whereas 76.66% of the PSA recurrence cases (PSA failure) had high Gleason grade [7(4+3) to 9].

**Figure 2:**
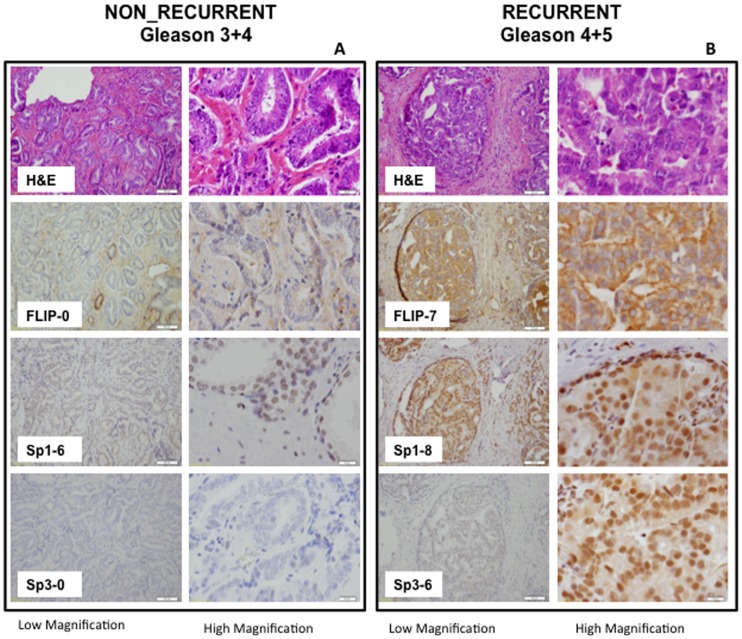
A. H&E staining and IHC analysis of expression of FLIP, Sp1, and Sp3 in a representative sample of non-recurrent PCA [Gleason 7 (3+4)] under low magnification (left) and high magnification (right). The total score for this sample was 0, 6, and 0 for FLIP, Sp1, and Sp3 respectively. **B.** H&E and IHC staining of FLIP, Sp1 and Sp3 in a representative sample from a patient with recurrent PCA [(Gleason 9 (4+5)] under low magnification (left) and high magnification (right). The total score for this sample was 7, 8, and 6 for FLIP, Sp1, and Sp3, respectively.

### Antibodies and Immunohistochemistry

Rabbit polyclonal antibodies specific for FLIP, Sp1, and Sp3 were from Santa Cruz Biotechnology (Santa Cruz, CA). Immunohistochemistry (IHC) was carried out in the pathology core facility of the Department of Pathology University of Texas Health Science Center at San Antonio. Staining was performed using standard IHC methods including the use of appropriate negative controls. Rabbit HRP polymer and DAB chromogen was used as the ancillary system and hematoxylin (DAKO North America Inc. Carpentaria, CA) was used for counterstaining.

**Figure 3:**
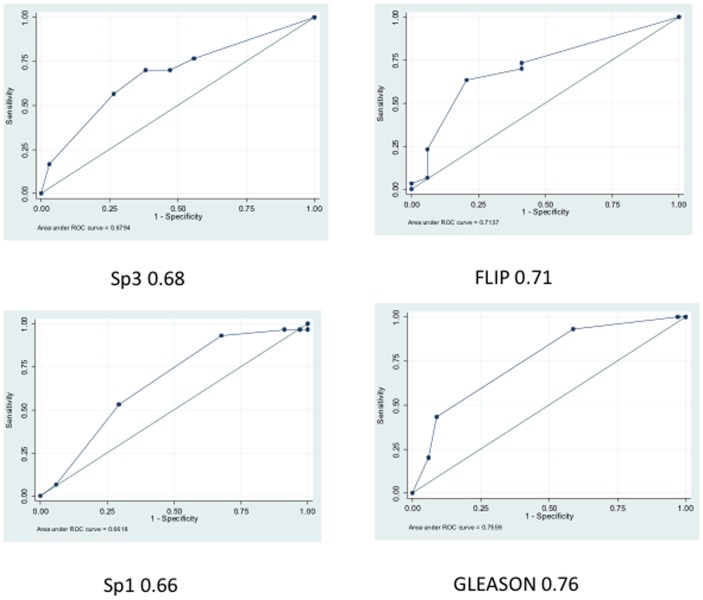
Plot of sensitivity versus specificity. Area under the ROC curves calculated for FLIP (0.71), Sp1 (0.66), Sp3 (0.68), and Gleason (0.76) show various degrees of discrimination as predictors of recurrence. An area under the ROC curve of 0.8 to 1.0 is considered to be very good to excellent discrimination, whereas 0.5 indicates no discrimination.

### Semiquantitative Evaluation of Tissue Staining

Tissue sections containing 30–40% tumor were chosen for pathological evaluation. A pathologist (I-TY) blindly evaluated staining of prostate tissue. Staining intensities and proportion of positive staining tumor cells were determined independently. Briefly, the proportion of positive tumor cells was scored as follows: 0, no stained cells; 1, ≤1%; 2, 1–10%; 3, 10–33%; 4, 33–66%; 5, 66–100% positive staining. The intensity score (IS) represents the average staining intensity of tumor cells: 0, no staining; 1, weak; 2, moderate; 3, strong staining. The proportion score and the intensity score were added to obtain the total score (TS) with a range of 0 to 8.

**Figure 4:**
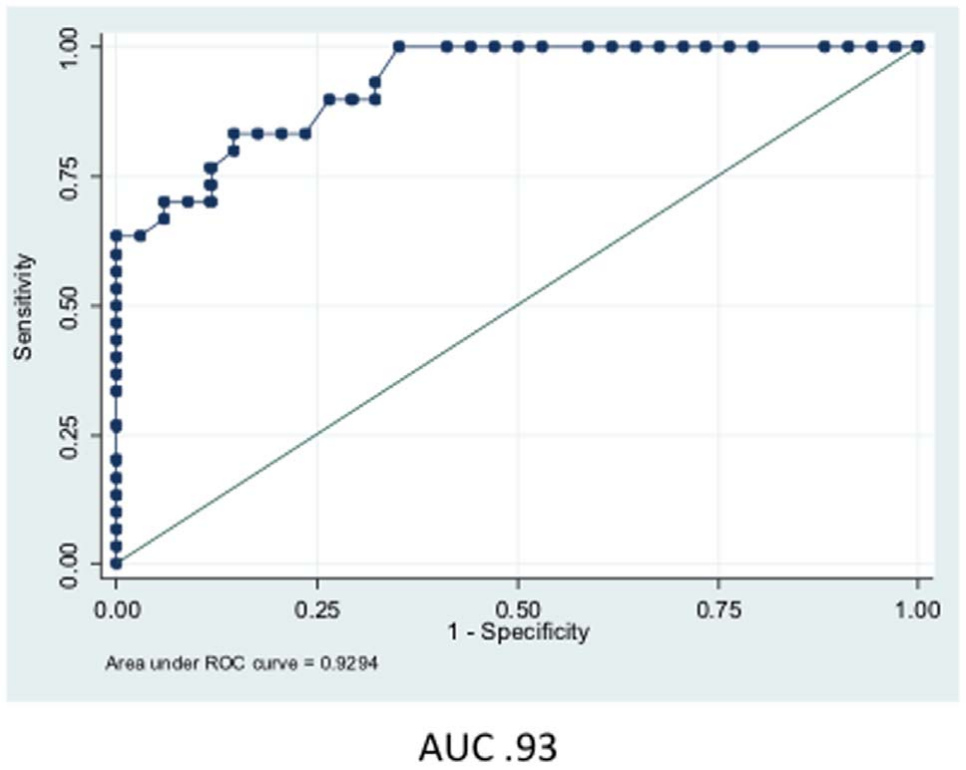
Plot of sensitivity versus specificity. Area under the ROC curves calculated for combination of FLIP, Sp1, Sp3, Gleason score, and their interactions gives a value of 0.93 indicating excellent discrimination between non-recurrent and recurrent cases.

### Statistical Methods and Analysis

Association of the FLIP/Sp1/Sp3 biomarker signature with clinical outcome (recurrence vs. non-recurrence) was evaluated using multiple statistical methods. The mean staining scores for protein expression in the two groups were compared with a Wilcoxon rank-sum test. p-values <0.05 were considered significant. The predictive value of each marker (FLIP, Sp1, and Sp3) for clinical outcome (recurrence or non recurrence) was first explored individually with logistic regression, and then the additive predicted value of the FLIP/Sp1/Sp3 signature and the extent to which they interacted with each other and with the Gleason score was explored with a backward selection model. The discrimination of the markers was identified with sensitivity-specificity analysis and the diagnostic value of the FLIP/Sp1/Sp3 signature was determined using area under the curve (AUC) for receiver operator characteristic (ROC) curves. Variables with p<0.15 were retained [14] in order to improve accuracy of the significant (p<0.05) variables reported. For the final model the Hosmer-Lemeshow Goodness of Fit test was performed. Significance levels and AUC for the ROC curve are reported. The analysis was carried out using SAS version 9.2 (SAS Institute Inc.) and STATA version 9.2 (STATA Corporation).

**Figure 5:**
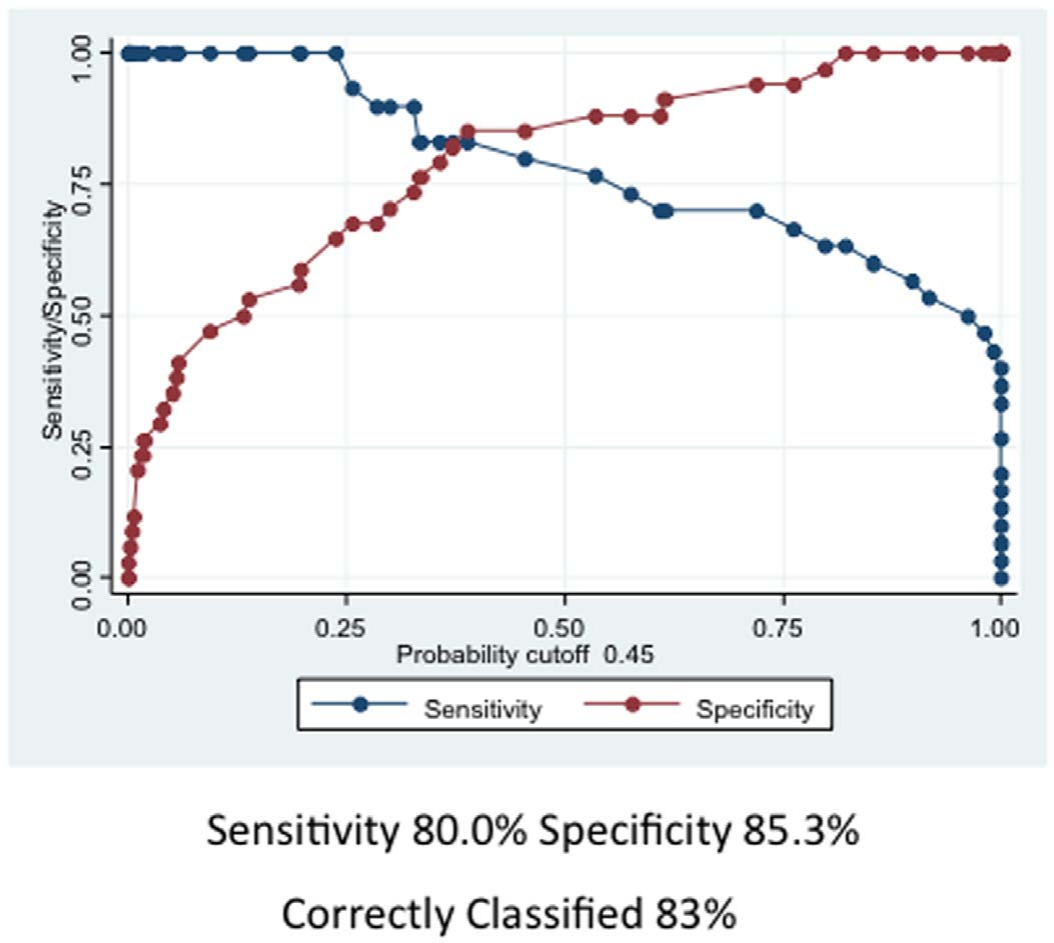
Plot of sensitivity versus specificity. At a probability cut-off point of 0.45 both the sensitivity (80%) and specificity (85.3%) for this combination of markers is high, indicating excellent discrimination power of the combination.

## Results

In this study we assessed the expression of the anti-apoptotic protein FLIP and its transcription regulators Sp1 and Sp3 by immunohistochemical evaluation of tissue samples obtained from 64 patients who underwent radical prostatectomy as primary treatment for prostate cancer. Patients had at least 60 months follow-up with PSA measurements and only those with an undetectable PSA at 60 months were considered to have non-recurrent disease. Increasing levels of PSA after prostatectomy were used as a surrogate endpoint for poor outcome. PSA non-failure was defined as PSA levels undetectable or <0.2 ng/mL for at least 5 years after prostatectomy and no other signs of recurrence such as metastasis. PSA failure was defined as a PSA level >0.2 ng/mL that increased during the 5 years after prostatectomy [15]. Due to limited sample size only two-way interactions were considered and PSA was not added to the Gleason score. First, we compared the expression of FLIP, Sp1, and Sp3 between the two groups using immunohistochemistry and found significant differences between PSA failure and non-failure groups in the expression of FLIP, Sp3, and Sp1 (Wilcoxon rank-sum; Figure 1 and Figure 2). As shown in the box plots in Figure 1, we found significant differences in the mean total IHC score between the non-recurrent and recurrent cases for Sp1 (p = 0.019), Sp3 (p = 0.011), and FLIP (p = 0.0019). We also included Gleason score in our analysis because this will have an influence on the outcome. Gleason scores for our 64-patient cohort were significantly different in the recurrent and non-recurrent groups (p = 0.0001; data not shown). It should be mentioned that this is not necessarily the case as studies have shown that Gleason grade 7 by itself may not be significant [16]. In our cohort, 50% of prostatectomy cases were Gleason 7: (29.69% were 3+4 and 20.3% were 4+3). Of the 29.69% that were 3+4, 41.2% were non-recurrent and 16.67% were recurrent cases. On the other hand, of the 20.3% with the more aggressive 4+3 grading, 8.8% were non-recurrent and 33.33% were recurrent. These data suggest that the differences in FLIP, Sp1, and Sp3 between the biochemically recurrent and non-recurrent groups are significant.

**Figure 6:**
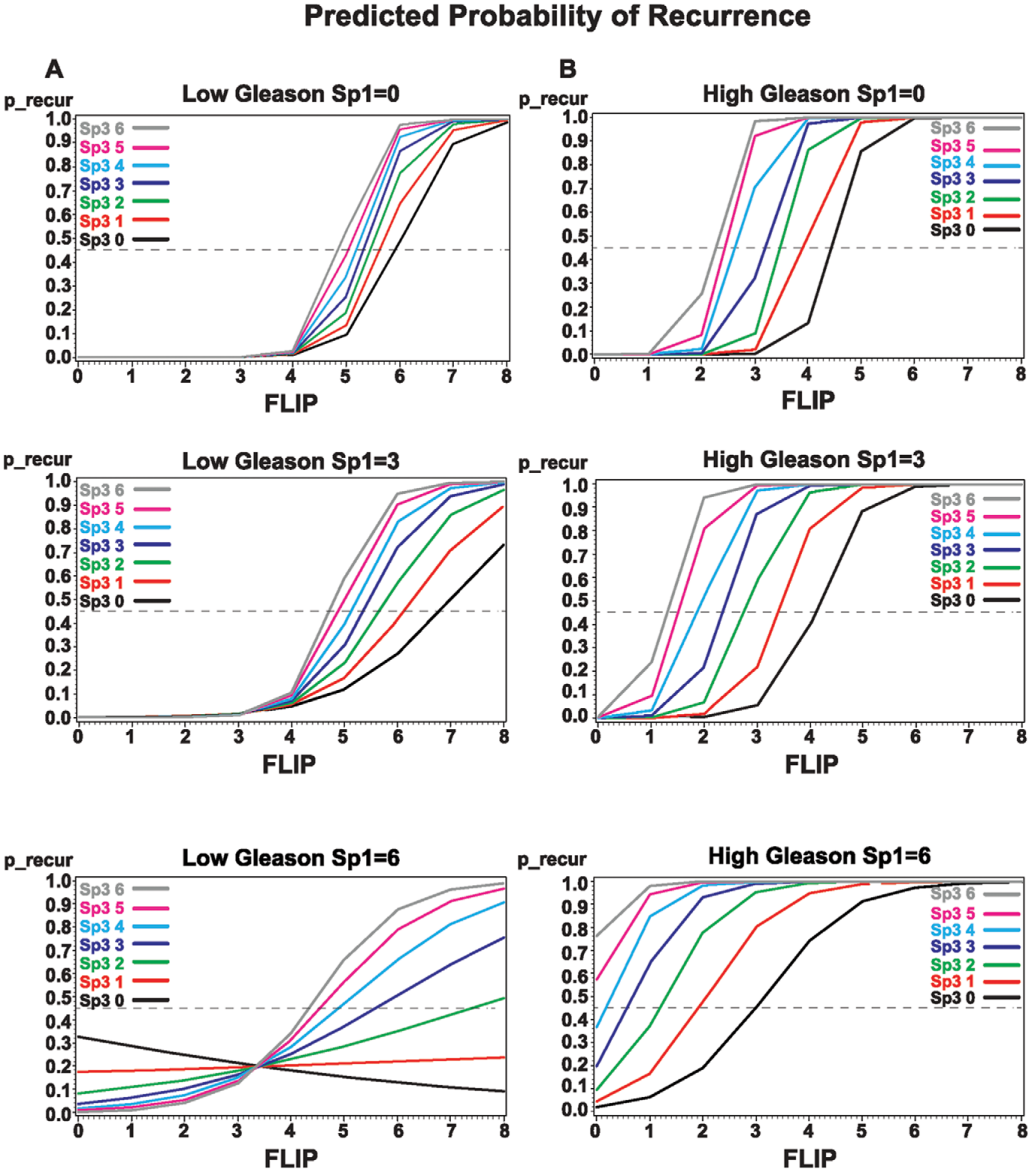
A. Predicted probability of recurrence when Gleason is low grade 5–7(3+4) for different levels of Sp1 (0, 3, and 6) and Sp3 (0–6) as a function of FLIP (0–8) interaction. Cases above the cut-off point of 0.45 (dashed line) are predicted to recur. The interaction of FLIP and Sp3 is shown as solid color lines on the X-axis. **B.** Predicted probability of recurrence when Gleason is high grade 7 (4+3) for different levels of Sp1 (0, 3, and 6) and Sp3 (0–6) as a function of FLIP (0–8) interaction. Cases above the cut-off point of 0.45 (dashed line) are predicted to recur. The interaction of FLIP and Sp3 is shown as solid color lines on the X-axis.

Based on the significant differences observed between biochemically recurrent and non-recurrent groups, we next calculated the sensitivity and specificity of the generated data [16]–[17]. Univariate logistic regression of FLIP, Sp1, Sp3, and Gleason grade resulted in AUCs for ROC curves of 0.71, 0.66, 0.68, and 0.76, respectively (Figure 3).

**Table 2:** Univariate analysis of odds ratios for the markers Sp1, FLIP, Sp3, and Gleason score (A) and multivariate analysis showing the main effects and their ability to predict recurrence in combination (B).

A-Univariate				B-Multivariate			
**Marker**	OR	P>|z|	95% CI	**Markers**	OR	P>|z|	95% CI
**Sp1**	1.4	0.162	0.87–2.25	**Sp1**	8.08	0.08	0.80–81.4
**FLIP**	1.43	0.005	1.1–1.84	**FLIP**	18.7	0.07	0.79–443.9
**Sp3**	1.3	0.018	1.05–1.65	**Gleason**	0.04	0.14	0.006–2.85
**Gleason**	3.3	0.001	1.6–6.7	Gleason* FLIP	4.3	0.03*	1.15–16.04
**Gleason gps.**	2.2	0.0001	1.47–3.29	Gleason* Sp3	5.55	0.01*	1.41–21.82
				Sp1* FLIP	0.67	0.11	0.41–1.1
				Sp3* FLIP	1.28	0.02*	1.04–1.58

Given the above results we explored the prognostic value of the markers using a multivariable logistic model with a backward selection that included Gleason score (high vs. low) (p = 0.14), FLIP (p = 0.07), and Sp1 (p = 0.08) as main effects and the interactions of FLIP with Sp3 (p = 0.02), Sp1 (0.11), and Gleason (p = 0.03), as well as the interaction of Sp3 and Gleason (p = 0.014), as second-term effects. These interactions were significantly different between non-recurrent and recurrent groups. Variables with p<0.15 were retained for the Hosmer-Lemeshow Goodness of Fit model, using PSA failure vs. non-failure as the dependent variable. The model showed a good fit, with chi-square value of 8.8 and p = 0.4, with an AUC for the ROC curve of 0.93 (Figure 4). At the optimum cut-off point of 0.45, the sensitivity was 80% and specificity was 85.29%, resulting in correct classification in 83% of the cases (Figure 5).

Figure 6 shows patients that are predicted to recur based on these biomarkers. Both Gleason and PSA alone have sensitivities below 80% therefore this model is an improvement on the markers currently in use. Our logistic regression prediction plot shows that a combination of FLIP, Sp1, and Sp3 in addition to Gleason is prognostic of PSA failure and non-failure. When we plot the model results for the predicted probability of recurrence on the Y-axis and the interaction of FLIP-Sp3 by Gleason grade (low or high) on the X-axis, we can clearly see the impact of interaction between the two markers and the influence of the Gleason grade, and also the influence of Sp1 at three levels (total score of 0, 3, and 6). In Figure 6 A & B, all cases above the cut-off point of 0.45 (dashed line) are predicted to be recurrent. With each increase in the staining score of Sp3, together with an increase in FLIP, the risk of recurrence goes up even with a low Gleason grade of 5–7 (3+4). However, when Gleason grade is high 7 (4+3)-9 and Sp1 is high (6), the risk increases dramatically. When FLIP is 4 (range 0–8) and Gleason grade is high, both Sp3 and Sp1 need to be near 0 for a case to be non-recurrent, but when the Sp1 score is 3, cases with a FLIP score of 4 are recurrent when Sp3 is ≥1 (Figure 6 A and B). This model shows that FLIP, Sp1, and Sp3 levels in conjunction with Gleason grade can be a good predictor of the risk of biochemical recurrence after radical prostatectomy. Figure 6 indicates that for a given value of Sp3 score (independent of Sp1 score), the predicted probability of recurrence increases with increasing FLIP staining when the Gleason score was low, suggesting potential interaction. On the other hand, when the Gleason score was high, although the predicted probability of recurrence increased with FLIP staining when the Sp1 score was 0 or 3, when the Sp1 score was 6, we did not see this interaction, suggesting that Gleason and Sp3 are sufficient for predicting recurrence (Table 2).

The data presented in this manuscript show the importance of the FLIP/Sp1/Sp3 signature for predicting biochemical recurrence in this cohort of patients. Our previously published studies demonstrated the importance of the transcription factors Sp1 and Sp3 in the transcriptional regulation of FLIP [13]. The current data indicate that these proteins are significant players in the recurrence of prostate cancer. Further gene silencing experiments using prostate cancer cell lines suggest that Sp1 physiologically regulate FLIP (data not shown). These results suggest that FLIP expression is positively regulated by Sp1 in tumor cells and that targeting Sp1/Sp3/FLIP could be a potential avenue for the clinical management of recurring prostate cancer. We speculate that Sp1/Sp3 could inhibit FLIP promoter activity, leading to activation of apoptotic signaling.

## Discussion

Effective clinical management of PCA has been hampered by significant intratumoral heterogeneity combined with an incomplete understanding of the molecular events associated with the development of the disease and subsequent recurrence following traditional treatments [18]–[19]. Therefore, there is an unmet need for new methods and/or agents for PCA management. Given the individual genetic variation and the heterogeneity of the disease, personalized treatment approaches are critical for successful management of PCA. To develop such individualized treatment approaches, it is essential to identify a panel of biomarkers or a “biomarker signature” that could be used to stratify patients according to response to specific treatments [20]–[21]. Although serum-based PSA screening is widely used, PSA has the following limitations as an early detection biomarker [20]–[23]: (i) Elevated levels of serum PSA have been observed not only in prostate cancer, but also in benign prostatic hyperplasia patients, therefore PSA is not specific to prostate cancer, and (ii) PSA is not sufficiently sensitive as indicated by the Prostate Cancer Prevention Trial (PCPT), which demonstrated that 15% of men with PSA levels of 4 ng/ml had prostate cancer and 15% of these patients had high Gleason grade disease. In addition, two randomized trials showed a modest effect of PSA screening on prostate cancer mortality, suggesting a substantial risk of negative biopsy and overdiagnosis and overtreatment of indolent cancer. Although numerous markers including α-methyacylCoA-racemase (AMCAR), fatty acid synthetase (FASN), ERG, and prostate-specific membrane antigen (PSMA), have been identified based on preclinical studies and shown to be associated with the outcome of prostate cancer after surgical treatment using human tissue samples, very few of these have predictive value independent of traditional prognostic factors such as Gleason score, pathological stage, and pretreatment PSA levels [5]–[6]. To the best of our knowledge, there are currently no sensitive markers to monitor disease recurrence.

In this study we assessed the expression of the anti-apoptotic protein FLIP and the transcription factors Sp1 and Sp3 by immunohistochemical evaluation of tissue samples obtained from 64 patients who underwent radical prostatectomy as primary treatment for prostate cancer. We believe that this is the first report of FLIP, Sp1, and Sp3 expression and the correlation among these proteins in biochemically recurrent PCA samples. Although increased expression of Sp1, Sp3, or FLIP showed significant differences between PSA failure and non-failure cases, individually they are not strong predictors of poor clinical outcome based on AUC when PSA failure is used as a surrogate outcome: the area under the ROC curve for FLIP, Sp1, Sp3, and Gleason as a predictor of PSA failure and non-failure cases was 0.71, 0.66, 0.68, and 0.76 respectively. On the other hand, the biomarker signature of Sp1/Sp3/FLIP combined with Gleason achieved an AUC of 0.93. These data indicate excellent discrimination between PSA failure and non-failure cases and suggest that this biomarker signature is an important predictor of the probability of biochemical recurrence. This is significant since current diagnostic procedures cannot distinguish between aggressive and clinically indolent disease, resulting in more men being treated for the disease than necessary. Our three-gene signature combined with Gleason grade was accurate 83% of the time in our cohort.

The observation that Sp1/Sp3 and FLIP may be predictors of clinical outcome could reflect their important role in prostate cancer. Increased levels of Sp1/Sp3/FLIP might be related to apoptotic resistance and progression to biochemical recurrence or progression from low- to high-risk prostate cancer. Cellular FLICE-inhibitory protein (c-FLIP) is a truncated form of caspase-8 that has been shown to play a critical role in the development of resistance to therapeutics in cancer cells by inhibiting apoptosis mediated by death receptor signaling [24]–[25]. Accordingly, FLIP is overexpressed in various cancers and this overexpression has been shown to determine therapeutic resistance [26]–[35]. In addition, overexpression of FLIP has been correlated with poor prognosis in colon, bladder, and urothelial cancers [26]–[35]. Recent studies from our laboratory demonstrated that specimens from high-grade prostate cancer exhibit higher expression of FLIP than those from low-grade tumors [13]. Furthermore, we also showed that FLIP is regulated transcriptionally through modulation of the transcription factors Sp1 and Sp3 and that inhibition of FLIP prevented prostate tumor development in a preclinical animal model [13]. Sp1 and Sp3 belong to the Zn-finger family of transcription factors that have been shown to regulate expression of genes involved in various cellular processes of oncogenesis including differentiation, apoptosis, cell migration, and cell cycle progression [36]–[38]. Sp1 and Sp3 have similar structural features including a highly conserved DNA binding domain and consequently bind to DNA with similar affinity. Although Sp1 is a known trans-activator, Sp3 functions both as an activator and as a repressor depending on the cellular context. Although studies on Sp3 and cancer are lacking, Sp1 levels have been shown to be elevated in a wide variety of cancers including breast, thyroid, hepatocellular, pancreatic, colorectal, gastric, and lung cancer [38]. Furthermore, abnormal Sp1 protein levels have been correlated with cancer stage and poor prognosis. Accordingly, inhibition of Sp1 or its knock-down to normal cellular levels usually decreases tumor formation, growth, and metastasis. It is noteworthy that we previously showed that Sp1 trans-activates FLIP in prostate cancer cells, whereas Sp3 inhibits this trans-activation [13]. Based on these data we expected to see an inverse association between Sp1 and Sp3 in these samples. However, the observed positive association suggests that Sp1 and Sp3 have a similar functional role in the context of the tumor microenvironment although other factors, such as the small sample size, could also contribute to these observations. Our data suggest that FLIP expression could be positively regulated by Sp1 in tumor cells and that targeting Sp1/Sp3/FLIP could be a potential avenue for clinical management of recurring prostate cancer.

This is the first report to describe a three-gene signature that might be used to assess whether a patient’s cancer will recur following a given therapy. Such a tool would have a significant impact on the clinical management of prostate cancer. Previous studies reported that AR and pAkt staining predicts recurrence after prostatectomy [17], [39] and it is possible that combining these markers with those of this study may further enhance prediction of recurrence. Larger follow up studies, including validation of these findings using independent data sets, are warranted to assess the usefulness of this biomarker signature. Replication of these results and the inclusion of all known prognostic factors in the study would also strengthen the validity of this biomarker signature, alone and in combination. In summary, our data indicate that the Sp1/Sp3/FLIP signature in combination with Gleason grade is predictive of recurrence of prostate cancer and that its clinical application might avoid unnecessary aggressive interventions, thus improving quality of life and reducing healthcare related expenses.

### Translational Relevance

Although prostate specific antigen (PSA) is widely used for the detection of prostate cancer, there is a need for a biomarker(s) that reliably predicts prostate cancer recurrence. We previously identified transcription factors Sp1 and Sp3 as regulators of the anti-apoptotic protein FLIP using cell culture models. Subsequent studies showed that inhibition of prostate tumor development in a preclinical animal model was accompanied by down-regulation of FLIP and Sp1. Here we investigated the potential use of these genes as predictors of prostate cancer prognosis. We retrospectively analyzed tissues from patients with recurrent and non-recurrent prostate cancer who had undergone radical prostatectomy as primary treatment and were followed for at least 5 years with PSA measurements. The areas under the receiver operating characteristic curves for individual markers FLIP, Sp1, Sp3, and Gleason score for prediction of PSA failure and non-failure were 0.71, 0.66, 0.68, and 0.76 respectively. This improved to 0.93 when the markers were combined with Gleason and their interactions were considered. Based on these analyses, we conclude that the “biomarker signature” of FLIP/Sp1/Sp3 can predict disease recurrence and stratify patients likely to benefit from more aggressive treatment. These results also provide support for targeting the Sp1/Sp3/FLIP axis for prostate cancer management and warrant further studies using a larger sample size and additional clinical data sets.
